# Clothing Insulation Rate and Metabolic Rate Estimation for Individual Thermal Comfort Assessment in Real Life

**DOI:** 10.3390/s22020619

**Published:** 2022-01-14

**Authors:** Jinsong Liu, Isak Worre Foged, Thomas B. Moeslund

**Affiliations:** 1 Visual Analysis and Perception Laboratory, CREATE, Aalborg University, 9000 Aalborg, Denmark; tbm@create.aau.dk; 2 The Royal Danish Academy—Architecture, Design, Conservation, 1435 Copenhagen, Denmark; hfog@kglakademi.dk

**Keywords:** thermal comfort, clothing insulation rate, metabolic rate, multi-person, real life

## Abstract

Satisfactory indoor thermal environments can improve working efficiencies of office staff. To build such satisfactory indoor microclimates, individual thermal comfort assessment is important, for which personal clothing insulation rate ($I_{cl}$) and metabolic rate (*M*) need to be estimated dynamically. Therefore, this paper proposes a vision-based method. Specifically, a human tracking-by-detection framework is implemented to acquire each person’s clothing status (short-sleeved, long-sleeved), key posture (sitting, standing), and bounding box information simultaneously. The clothing status together with a key body points detector locate the person’s skin region and clothes region, allowing the measurement of skin temperature ($T_{s}$) and clothes temperature ($T_{c}$), and realizing the calculation of $I_{cl}$ from $T_{s}$ and $T_{c}$. The key posture and the bounding box change across time can category the person’s activity intensity into a corresponding level, from which the *M* value is estimated. Moreover, we have collected a multi-person thermal dataset to evaluate the method. The tracking-by-detection framework achieves a mAP_50_ (Mean Average Precision) rate of 89.1% and a MOTA (Multiple Object Tracking Accuracy) rate of 99.5%. The $I_{cl}$ estimation module gets an accuracy of 96.2% in locating skin and clothes. The *M* estimation module obtains a classification rate of 95.6% in categorizing activity level. All of these prove the usefulness of the proposed method in a multi-person scenario of real-life applications.

## 1. Introduction

In the world today, more people have to rely on computers to tackle various tasks. This results in indoor office work being much more popular than ever before. From the commercial buildings energy consumption survey in 2012 [1], offices consume much more energy for heating and cooling than other types of buildings. If energy can be used according to office workers’ thermal needs, energy waste resulting from overheating or overcooling will be greatly reduced, and also staff will have better working efficiencies as they feel comfortable with the environment they work in.

To make each office staff feel thermal comfort and at the same time reduce energy waste, two main kinds of methods have been researched. One is directly relying on the worn clothes to control a person’s micro-environment between the body skin and the indoor atmosphere, which avoids controlling the entire indoor microclimate via heaters, ventilation, and air conditioners (HVAC) that consume lots of energy. This kind of method takes advantage of different thermal properties (thermal resistance, thermal conductivity, thermal radiation, thermal convection, water evaporation, etc.) of different clothes in materials, thicknesses, and layers to maintain the body temperature in a comfortable range [2,3,4,5]. The other kind of method still focuses on the entire indoor environment but in a way that adjusts the microclimate according to each occupant’s thermal need, which is the topic of this paper.

However, each person’s thermal need is unique and dynamic, which cannot be met well by existing microclimate-controlling systems like HVAC that all rely on static assumptions to serve the occupants in a room. For example, a standard air conditioner’s temperature is set to 25 to 27 degrees for cooling in summer, and 18 to 20 degrees for heating in winter, no matter whether this is what the office workers need.

To improve this situation, individual thermal comfort feeling has to be assessed, like in scales (cold, cool, slightly cool, neutral, slightly warm, warm, and hot) [6,7,8]. These scales depend on both environmental factors and personal factors. The environmental factors are air temperature ($t_{a}$), mean radiation temperature (${\bar{t}}_{r}$), relative humidity (RH), and air velocity ($V_{a}$), which can be measured by sensors. The personal factors include clothing insulation rate ($I_{cl}$) and metabolic rate (*M*); $I_{cl}$ describes the ability of the clothes to insulate the heat exchange between the skin and the environment outside the clothes, and *M* describes the amount of energy, in-unit time, consumed by a person. Both the personal factors are difficult to acquire for their complexity and dynamics.

Accordingly, international standards [8,9,10,11,12] have defined reference values of $I_{cl}$ and *M* in certain situations (see Table 1 and Table 2). Such values are empirical and fixed, and thus cannot describe a person’s dynamic property for that the situation in real life is much more complex than these noted ones. This hinders the development of systems and applications for adjusting indoor microclimates according to occupants’ thermal needs. Therefore, the solution dynamically estimating a person’s $I_{cl}$ and *M* is to be explored. To this end, we propose a method to do this, and the concrete contributions are:

- The method inventively adapts state-of-the-art computer vision solutions to the thermal comfort domain, achieving a contactless approach that can be employed in multi-person real-life applications.
- The method can detect and track each person, at the same time recognizing his or her clothing status (long-sleeved, short-sleeved) and key posture (sitting, standing).
- The method can further output a person’s skin temperature and clothes temperature, based on which his or her $I_{cl}$ is estimated.
- The method proposes three useful features from a person’s bounding box tracked across time. These features can category the person’s activity into a certain intensity level which indicates the *M*.

The rest contents are organized as follows. Section 2 introduces the related work. Section 3 describes our methodology. Section 4 tells the experiments. Section 5 concludes the paper and proposes future work.

## 2. Related Work

This paper applies computer vision solutions to the thermal comfort domain. Therefore, the related researches of both $I_{cl}$ and *M* estimation and computer vision methods are studied.

### 2.1. $I_{cl}$ and *M* Estimation

Several works have been published to calculate the two personal factors, $I_{cl}$ and *M*, for assessing the human thermal sensation. However, most works only focus on one of them, leaving the other one unsolved.

Some works take advantage of the relationship between clothing choice and environment temperature [13,14,15,16] to predict clothing insulation ability. This type of method is simple but neglects the inherent property of clothes themselves. To resolve this drawback, work [17] uses the weight of the clothes to predict $I_{cl}$, which is unrealistic in real applications; studies estimate $I_{cl}$ from the temperature difference between the body skin and the clothes surface with infrared sensors [18,19], however, this is also inconvenient due to the attached sensors on the human body. To decouple such interference with personal life, researches [20,21,22] all adopt contactless infrared cameras to monitor persons. Unfortunately, refs. [20,21] do not mention the method of acquiring temperatures of interested body locations, limiting their applications in the real world; ref. [22] only considers five types of garments that cannot represent various clothing choices in daily life.

For metabolic rate estimation, almost all works have to use attached equipment. Correspondingly, a person’s *M* is estimated by measuring his or her oxygen consumption and carbon dioxide generation [23,24,25], heart rates [26,27,28,29], or blood pressure [30]. Though [31,32,33] adopt cameras for such a task, they still partly rely on sophisticated equipment mentioned above. These devices have to be worn by subjects, making them unrealistically used in daily life.

When estimating both $I_{cl}$ and *M*, refs. [34,35] use a CNN (Convolutional Neural Network)-based classifier to recognize a person’s clothes type and activity type, and then refer ISO (International Organization for Standardization) standard tables to get the $I_{cl}$ and *M* values from the recognized types. These works prove the importance of clothing status (short sleeves, long sleeves) and posture (sitting, standing) in estimating $I_{cl}$ and *M*. However, refs. [34,35] are only valid in a simple and controlled single-person environment. Expanding and enriching this kind of solution is in great need. Therefore, this paper closes this gap and is the first work targeted at a multi-person scenario in the real world.

### 2.2. Detection and Tracking

The ability to do individual processing from multiple persons is the crucial point of the proposed method, which mainly comes from our implemented human tracking-by-detection framework. To this end, widely used object detectors are studied, like Faster R-CNN (Region-based Convolutional Neural Network) [36], YOLO (You Only Look Once) series [37,38,39,40,41], and FPN (Feature Pyramid Network) [42] which all consist of a backbone network (to extract deep features) and headers (to predict bounding box locations and categories). All these methods perform well on RGB (Red Green Blue) benchmark datasets [43,44].

When it comes to the tracking part (referring in particular to online multi-object tracking in this paper), SORT (Simple Online and Realtime Tracking) [45] initially replaces the conventional object detector with a CNN-based detector and thus improves the tracking result by up to 18.9%,revealing the importance of accurate detections for tracking. The following DeepSort (Simple Online and Realtime Tracking with a Deep Association Metric) [46] and CDA_DDAL (Confidence-based Data Association and Discriminative Deep Appearance Learning) [47] incorporate appearance information into the data association phase and solve the ID (Identity)-switch problem. Other works focus on improving the correlation filter to estimate better positions of targets in the next frame [48], fusing multi-modality data in data association [49], and linking detection and tracking to let them benefit each other [50].

In general, though existing methods on human detection and tracking are quite mature in RGB datasets, studies applying them in thermal datasets like [51,52,53] are few and far between. This situation makes our research with the thermal camera more essential.

## 3. Methodology

**Figure 1:**
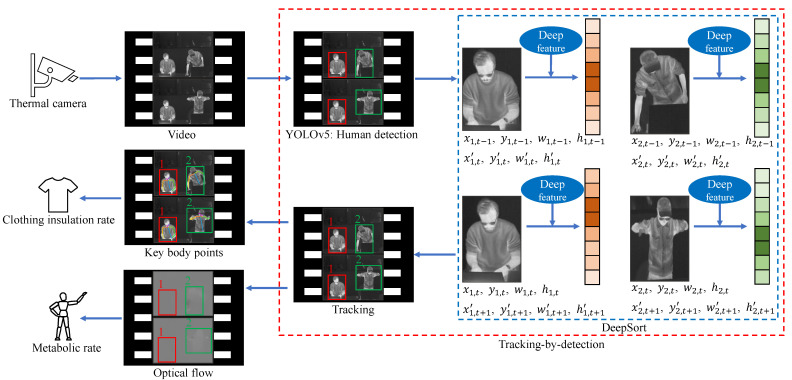
Overview of the proposed method. The numbers 1 and 2 are the corresponding tracking ID numbers of the two persons.

In this section, we describe our approach, the overview of which is illustrated in Figure 1 including three key parts:

1. The thermal input goes through a tracking-by-detection framework (see the red dashed box) to track each individual (see the ID 1 and ID 2) and at the same time categorize each person to get his or her clothing status and key posture (see the red and green solid boxes around persons which indicate different categories).
2. With ID information, for each person, the clothing status classified by the tracking-by-detection part helps differentiate the skin region from the clothing-covered region. Then the detected key body points from these two regions can represent the skin temperature and the clothes temperature, based on which $I_{cl}$ is estimated.
3. With ID information, for each person, the optical flow within each person’s bounding box region, together with the bounding box (center location and box size) changes across time are calculated. These three features are good representations of the person’s activity intensity, which are used to estimate *M*.

Details of the three parts are described below.

### 3.1. Tracking-by-Detection

This part has two main components, one is an object detector, YOLOv5 [41], for human detection, the other is a tracker, DeepSort [46].

**Figure 2:**
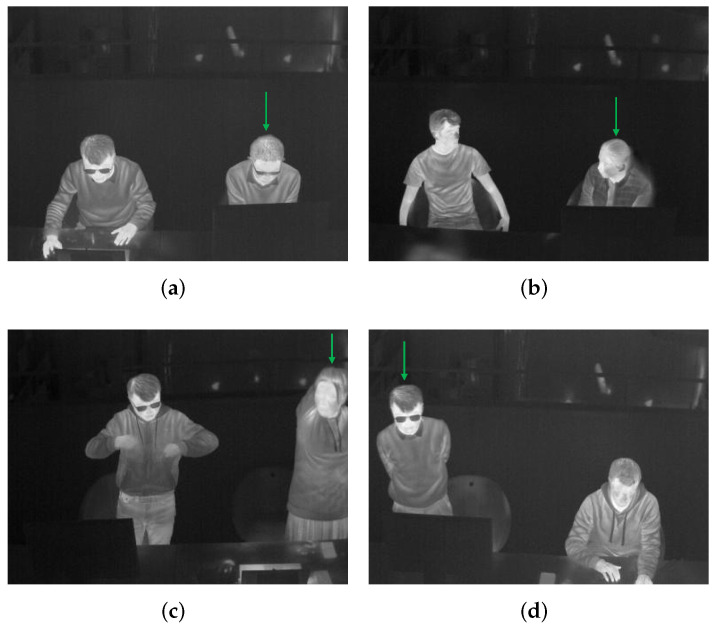
Persons difficult to predict clothing type due to occlusion. They are pointed by the green arrows. (**a**) The right person is partly occluded by the monitor. (**b**) The right person is partly occluded by the monitor. (**c**) The right person moves the arms out of the scene. (**d**) The left person hides the arms behind the torso.

The video collected from a thermal camera is the input to the detector YOLOv5 for frame-by-frame human detection. To integrate clothing status and key posture recognition into this detection procedure, we classify persons into six categories (see Table 3). Here the clothing status is represented by the sleeve status (long, short) for three reasons: (i) these two are the most common clothing situations in an office environment while the lower part of the body is often totally occluded by the desk; (ii) according to to [10,34,35], sleeve status is significantly important in estimating $I_{cl}$; (iii) the change between a long-sleeved status to a short-sleeved status by rolling up sleeves or taking off outer jackets is a sign of feeling hot and vice versa, indicating a person’s thermal sensation directly; (iv) the sleeves status helps to locate skin region and clothes region separately for further skin and clothes temperatures acquisition. For example, the elbows of a person wearing short-sleeved clothes are skin regions, while the elbows of a person wearing long-sleeved clothes are clothes regions. This localization makes it possible to use such key body points to calculate a person’s skin temperature and clothes temperature, because key body points on arms are widely used sensitive heat receptors in thermal comfort assessment [35,54,55,56]. Besides the two statuses of long sleeves and short sleeves, another status called difficult to predict clothes type due to occlusion is also usual in daily life. For clear illustration, such cases are in Figure 2. The right persons in Figure 2a,b are partly occluded by the computer monitor; the right person in Figure 2c moves the arms out of the scene; the left person in Figure 2d occludes his lower arms by hiding them behind the torso. These occlusions make it unrealistic to know whether the sleeves are long or short. One thing to be noted is that even though a person is occluded in a few frames, his or her clothing status can be recognized in other frames. Therefore, voting of a classified category over a few seconds is important. When it comes to the key posture recognition, from ISO standards [8,9,11,12], a person’s metabolic rate *M* is closely related to the behaving posture (sitting, standing, lying down, etc.). And in a typical office environment the most common ones are sitting and standing, therefore, these two are considered in our study.

The ultimate goal of this research is to acquire every occupant’s personal factors and thus facilitate individual thermal comfort assessment. This means that each person must be tracked across time. To this end, we adopt DeepSort. This tracker receives the image information and YOLOv5-predicted detections, and then decides which tracking ID a detection should be associated to. Like Figure 1 shows, DeepSort can use the detected bounding box information in the (t−1)th frame ($x_{i,t-1},y_{i,t-1},w_{i,t-1},h_{i,t-1}$ indicating the *i*th box’s top-left coordinates, width, height, respectively) to infer the location of the same object in the *t*th frame in the form of $x_{i,t}^{\prime },y_{i,t}^{\prime },w_{i,t}^{\prime },h_{i,t}^{\prime }$ by Kalman filter. At the same time, DeepSort extracts and saves the deep features of the object as its appearance information. In this way, two similarity metrics (location and appearance) can be calculated, based on which each detected person can be linked to a specific identity thus making the same person be tracked with a consistent ID over time.

The reason why this DeepSort-by-YOLOv5 paradigm is chosen and applied to such a specific research field is explained further below. The data we use is in a thermal mode having significantly fewer details compared with its RGB counterpart. This makes the reuse of such limited details/features extremely important. Compared with other detectors, YOLOv5 introduces PANet (Path Aggregation Network) [57] as its neck, making the deeper layers access to the lower-layer features much more efficiently, so the thermal features are well reused. When it comes to the tracking part, the Maximum Age strategy in DeepSort that deletes a track only when it is not associated to any detection more than $A_{max}$ frames can guarantee a consistent ID with the existence of a few false negatives (FN) from YOLOv5. The Tentative Track strategy in DeepSort which confirms a track only after it is associated with detection in three continuous frames also guarantees that occasional false positives (FP) from YOLOv5 have no severe influence on the output. That is to say, this tracking-by-detection framework smooths the direct output from a detector by filtering the undesired consequences of FN and FP, making both the detector and the tracker benefit each other. Additionally, the low complexity and real-time performance of DeepSort fit well the relatively simple scene in our case compared with other cases like pedestrians/vehicles tracking in autonomous driving assistance systems.

Overall, this design not only locates and tracks each individual with a consistent ID in the scene, but also predicts the person’s clothing and posture status simultaneously that directly influence $I_{cl}$ and *M* estimation.

### 3.2. $I_{cl}$ Estimation

$I_{cl}$ estimation relying on lookup tables in ISO standards [8,9,10,12] and updated clothes databases [58,59] can be a fast solution for laboratory studies, but it is unfeasible to use such a scheme in real applications due to reasons: (i) looking up the $I_{cl}$ value for a person needs extra manual work which is tedious and expensive; (ii) if this look-up task is expected to be done automatically, the solution must have the ability to recognize hundreds of different garment combinations that vary in materials and number of layers as the latest research has revealed the significant importance of them in thermal comfort [2], which is far beyond the capability of existing algorithms.

Therefore, to realize automated estimation, we go another way—using the difference between the skin temperature $T_{s}$ and the clothes temperature $T_{c}$ to calculate $I_{cl}$. This method is intuitive since the difference between $T_{s}$ and $T_{c}$ explicitly reveals the heat insulation of clothes to isolate the bare skin from the environmental air. The larger the temperature difference, the higher the clothing insulation rate.

**Figure 3:**
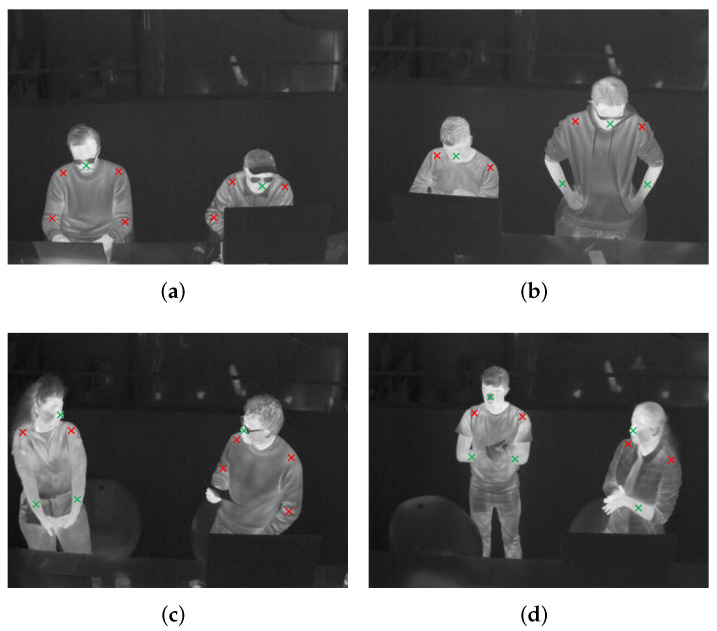
Skin region $R_{s}$ and clothing-covered region $R_{c}$. $R_{s}$ in green crosses and $R_{c}$ in red crosses. (**a**–**d**) illustrate persons doing different tasks in different poses.

To get $T_{s}$ and $T_{c}$ for each individual, the person’s skin region $R_{s}$ and clothing-covered region $R_{c}$ need to be differentiated from each other. Empirically, $R_{s}$ includes face, hands, and neck; $R_{c}$ includes shoulders, torso, and upper arms. However, in daily life, accessories (hat, glasses, scarf, watch, etc.), spontaneous behaviors (lower one’s head, turn one’s face away, hide one’s arm behind the torso, etc.), and inevitable occlusions by things in front make many body parts be detected unreliably and even totally invisible. After considering such situations, this research counts the lower arms (the middle point of the elbow and wrist) for short-sleeved clothes and the nose area as $R_{s}$, and the elbows for long-sleeved clothes and the shoulders as $R_{c}$. These regions are also widely used heat receptors in thermal comfort research [35,54,55,56]. Figure 3 illustrates $R_{s}$ in green crosses and $R_{c}$ in red crosses on four images.

**Figure 4:**
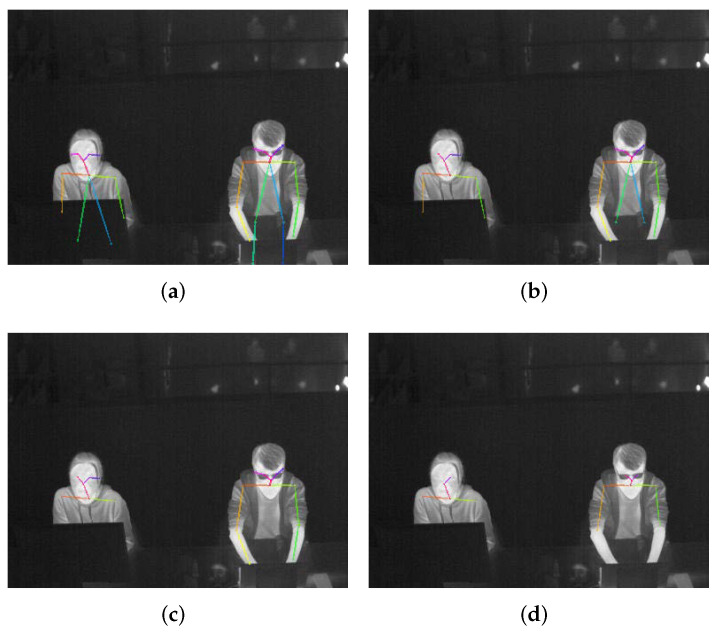
Detected key body points by OpenPose with different confidence thresholds, (**a**) threshold of 0.1, (**b**) threshold of 0.3, (**c**) threshold of 0.5, (**d**) threshold of 0.7. For better visualization, each key point is the end of the colorful line segment.

To locate these body parts, we employ OpenPose [60]—a 2D pose estimation tool. OpenPose has a robust ability against occlusions to detect key body points. The level of the ability against occlusions is determined by a parameter called confidence threshold which means that only the detected key point whose confidence score is higher than the threshold will be counted as the output. The higher threshold, the lower the level of ability against occlusions but the higher accuracy of detection; the lower threshold, the higher-level ability against occlusions but more false positives. This can be shown in Figure 4 which draws the detected key body points by OpenPose with different confidence thresholds of 0.1, 0.3, 0.5, and 0.7.

Since the detected key points are representations of $R_{s}$ and $R_{c}$ and thus directly related to $T_{s}$ and $T_{c}$, a higher accuracy instead of the ability against occlusions is much more important. Like in Figure 4a,b, the detected elbows of the left person are in fact in the computer monitor region; the result in Figure 4c is more accurate, but the detected wrists of the right person are in the laptop region which will influence the lower arm localization in $R_{s}$. These preliminary trials inspire us to set the confidence threshold as high as possible, but a too high threshold produces more missing detections. Therefore, our work uses 0.6 as the threshold in the entire research which has been proved as an effective parameter in the experimental part Section 4.3. To further decrease the influence of miss detections, an accumulation strategy of all the detected key points within a duration like five minutes is introduced since a person’s clothes status is not changed very frequently, which at the same time filters out potential noises.

Another thing worth mentioning is that although OpenPose detects key body points for each person, it has no function of multi-person tracking, and hence our tracking-by-detection framework is still necessary.

In mathematics, based on the recognized sleeves status and OpenPose-predicted key body points, the skin region $R_{s}$ and the clothing-covered region $R_{c}$ are determined, both of which are a set of pixel coordinates (x,y) in the image plane like Equation (1) and (2).

(1) $$R_{s}=\left\{\left(x_{t}^{1_{s}},y_{t}^{1_{s}}\right),\left(x_{t}^{2_{s}},y_{t}^{2_{s}}\right),\ldots ,\left(x_{t+1}^{m_{s}},y_{t+1}^{m_{s}}\right),\ldots ,\left(x_{t+itv-1}^{n_{s}},y_{t+itv-1}^{n_{s}}\right)\right\}$$

(2) $$R_{c}=\left\{\left(x_{t}^{1_{c}},y_{t}^{1_{c}}\right),\left(x_{t}^{2_{c}},y_{t}^{2_{c}}\right),\ldots ,\left(x_{t+1}^{m_{c}},y_{t+1}^{m_{c}}\right),\ldots ,\left(x_{t+itv-1}^{n_{c}},y_{t+itv-1}^{n_{c}}\right)\right\}$$

In the equations, the subscript (*t*, t+1, t+itv−1) refers to the index of each frame within a time period of itv frames; the superscript ($1_{s}$, $2_{s}$, $m_{s}$, $n_{s}$, $1_{c}$, $2_{c}$, $m_{c}$, $n_{c}$) refers to the index of each detected key point. So in the consecutive itv frames there are $n_{s}$ and $n_{c}$ key points detected in $R_{s}$ and $R_{c}$, respectively.

The thermal camera we use is Xenics Gobi-384-GigE that can visualize a thermography of the scene it captures and measure the temperature of each pixel within the image with an accurate resolution of 0.08 °C. Therefore, temperatures of the detected key points ($T_{1_{s}}$, $T_{2_{s}}$,..., $T_{n_{s}}$) in $R_{s}$ and ($T_{1_{c}}$, $T_{2_{c}}$,…, $T_{n_{c}}$) in $R_{c}$ are easily read from the camera. Then an average calculation of the temperature values ($T_{1_{s}}$, $T_{2_{s}}$,…, $T_{n_{s}}$) and ($T_{1_{c}}$, $T_{2_{c}}$,…, $T_{n_{c}}$) gets $T_{s}$ and $T_{c}$, respectively.

As long as $T_{s}$ and $T_{c}$ of each individual are calculated, the person’s $I_{cl}$ can be estimated by:

(3) $$I_{cl}=\frac{1}{0.155\;\cdot \;h}\;\cdot \;\frac{T_{s}-T_{c}}{T_{c}-T_{o}}$$

where *h* equals to 8.6 referring to human’s heat transfer coefficient; $T_{o}$ is the operative temperature considering both the air temperature and the mean radiation temperature, so here it is calculated by the average temperature of the background region in each frame. This calculation comes from [35] according to [10,61], and all the temperatures $T_{s}$, $T_{c}$, and $T_{o}$ are in degrees Celsius. We claim that our emphasis is the OpenPose strategy for localizing $R_{s}$ and $R_{c}$ to get $T_{s}$ and $T_{c}$, based on which any $I_{cl}$ calculation method can be applied.

### 3.3. M Estimation

In this part, we first in Section 3.3.1 propose three vision-based features to represent each person’s activity intensity, based on which *M* is estimated in Section 3.3.2.

#### 3.3.1. Three Vision-Based Features

Though *M* can be estimated by a person’s key posture or activity type listed in ISO standards [8,9,11,12] and updated databases [62,63], this is a rough estimation in many cases, since we have observed that different people tend to have different activity intensities for the same posture. For example, some people will do a bit of stretching when standing up while others may just stand still. Therefore, a more accurate and dynamic *M* estimation is expected. This is done by computing three vision-based features—a person’s bounding box changes in two aspects (location and scale) and the optical flow intensity within the bounding box, over a few seconds like 10 s (210 frames) in our case. Here, the choice of 10 s comes from an observation that it takes similar durations for a smart bracelet to monitor a user’s heartbeats and blood oxygen content—two human physiological signals indicating the *M* value. This three-feature idea is motivated by that: the bounding box location change captures the general body movement; the bounding box scale change captures the motion of limbs; the optical flow intensity within the box captures the subtle movement that the box changes may ignore.

To realize this, for the location change of a certain person’s bounding boxes during 10 s (210 frames), the center coordinates $\left(c_{x},c_{y}\right)$ of the person’s bounding box in each frame is drawn as a point in a 2D plane, and totally the 210 2D points form a cluster-shaped pattern. The more spread out the points are, the larger the general body movement is. The degree of spread can be approximated by fitting an ellipse to the cluster and then calculating the area of this ellipse. In mathematics, first, the covariance matrix of the vector $V_{cx}$ (composed of the horizontal coordinates of the 210 points) and the vector $V_{cy}$ (composed of the vertical coordinates of the 210 points) is computed, and then the two eigenvalues of the covariance matrix are computed, at last, the multiplication of these two eigenvalues represents the area of the ellipse.

For the scale change of a certain person’s bounding boxes, after translating the 210 bounding boxes from 210 frames, they will have the same center at the origin, and then the upper-right coordinates $\left(u_{x},u_{y}\right)$ of each bounding box represents its scale. Similarly, the 210 upper right points form a cluster in a 2D plane, and the area of the ellipse fitting to the cluster will represent the scale change across time. The larger the area, the larger movement of limbs.

When it comes to the optical flow intensity in a person’s bounding box from the *t*th to (t+itv−1)th frame (itv equals to 210 here), for each frame two optical flows in horizontal and vertical directions are extracted by the TV-L1 algorithm [64] realized in a tool called MMAction [65]. Each optical flow is saved as an 8-bit image in which pixels with a grayscale value of 127 represent no movement while these pixels with grayscale values farther away from 127 represent larger movements. Therefore, within a duration of itv frames, a person’s optical flow intensity $I_{xy}$ is calculated by:

(4) $$I_{xy}=\frac{\sum_{\tau =t}^{\tau =t+itv-1}I_{xy}^{\tau }}{itv}$$

(5) $$I_{xy}^{\tau }=\sqrt{{\left(I_{x}^{\tau }\right)}^{2}+{\left(I_{y}^{\tau }\right)}^{2}}$$

(6) $$I_{x}^{\tau }=\frac{\sum_{\left(x,y\right)\in box_{\tau }}^{}\left|f_{hrz}^{\tau }\left(x,y\right)-127\right|}{\sum_{\left(x,y\right)\in box_{\tau }}^{}1}$$

(7) $$I_{y}^{\tau }=\frac{\sum_{\left(x,y\right)\in box_{\tau }}^{}\left|f_{vtc}^{\tau }\left(x,y\right)-127\right|}{\sum_{\left(x,y\right)\in box_{\tau }}^{}1}$$

where τ indicates the frame index; $I_{xy}^{\tau }$ is the person’s optical flow intensity in the τth frame; $I_{x}^{\tau }$ and $I_{y}^{\tau }$ are the person’s optical flow intensity in the horizontal and vertical directions in the τth frame, respectively; (x,y) is any pixel in the optical flow; $box_{\tau }$ is the bounding box region of the person in the τth frame; $f_{hrz}$ and $f_{vtc}$ mean the two optical flows in the horizontal and vertical directions, respectively. In Equations (6) and (7), the number of pixels in the bounding box is acted as the denominator to normalize the influence of the size of the box.

**Figure 5:**
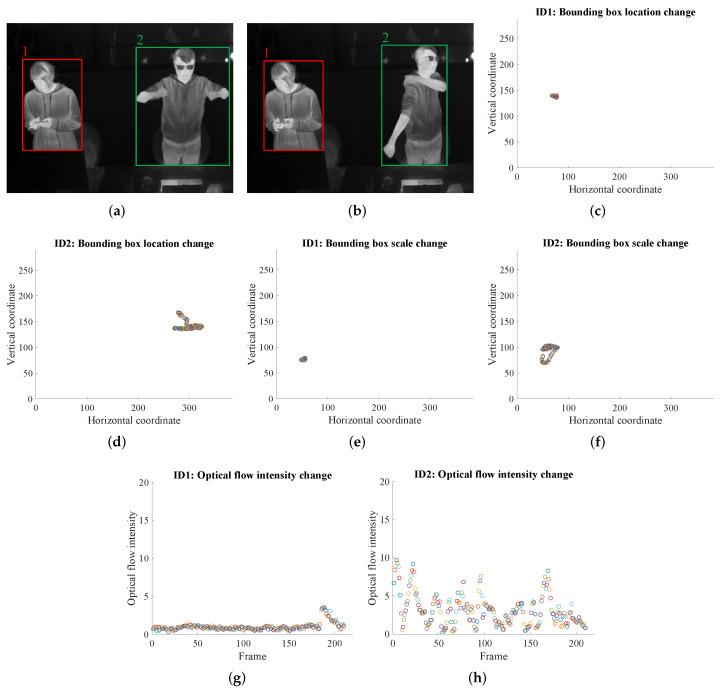
Using bounding box changes in location and scale, and the optical flow in the bounding box to represent an individual’s activity intensity. (**a**,**b**) ID 1 person is with small movements and ID 2 person is with large movements. (**c**,**d**) Bounding box location change of ID 1 person and ID 2 person, respectively. (**e**,**f**) Bounding box scale change of ID 1 person and ID 2 person, respectively. (**g**,**h**) Optical flow intensity change of ID 1 person and ID 2 person, respectively.

In this way, the three features (bounding box location change, bounding box scale change, optical flow intensity) representing an individual’s activity intensity are acquired. A visualization showing the bounding box location change by a cluster of 210 2D points/circles, the bounding box scale change also by a cluster of 210 2D points/circles, and the optical flow intensity within the bounding box in each frame from a duration of 210 frames are in Figure 5, in which ID 1 person is standing with very limited movements while ID 2 person is standing and stretching with large movements. This figure intuitively illustrates that the larger body movements of ID 2, the more spread out the points/circles in Figure 5d,f, and the larger optical flow intensity in Figure 5h.

#### 3.3.2. *M* Estimation from the Three Features

In real life, persons may have various activities which are unrealistic to be analyzed accurately. However, for an office environment, staff usually have scheduled routines and thus relatively fixed behaviors. Generally, the sitting staff are typing the keyboard, reading, taking notes, sorting through files, chatting with colleagues, online meetings, etc. And the standing staff are also occupied by the same tasks but may be involved with some walking or body stretching. This prior knowledge is such important that it gives a metabolic rate range from which each individual’s *M* varies.

Therefore, with the above prior knowledge of standard office behaviors, by referring Table A.1 and Table A.2 in ISO 8996 [11], the CBE (Center for the Built Environment) thermal comfort tool [66], and the 2011 compendium of physical activities tables [63,67], the usual metabolic rate range of a sitting office staff is quite narrow from 58 W/m^2^ (1.0 MET) to 87 W/m^2^ (1.5 MET), while a standing staff’s metabolic rate usually varies from 75 W/m^2^ (1.3 MET) to 174 W/m^2^ (3.0 MET). According to the CBE thermal comfort tool, the slight *M* change of a sitting person within the range [58 W/m^2^, 87 W/m^2^] has a mild influence on his or her thermal sensation, while the *M* change within the much larger range of a standing person significantly influences the thermal feeling. This result inspires us to use a middle value of 72.5 W/m^2^ to represent a sitting office staff’s *M* for simplicity and generalization which also relieves the three-feature extraction for him or her, but we need to specifically define a standing person’s *M* from his or her dynamic activity intensity situation represented by the three vision-based features.

To map such features to a value of *M*, a classification idea is introduced. Similar to Table A.2 in ISO 8896 where metabolic rates from 55 W/m^2^ to more than 260 W/m^2^ are categorized into resting, low, moderate, high, and very high levels, we decide to categorize the metabolic rate of a standing office staff into low, moderate, and high levels. Specifically, a low level means standing with very limited movements or transient spontaneous movements (standing quietly in a line, reading, using a cellphone, normally chatting, etc.); a moderate level means standing with spontaneous but lasting movements (natural and small paces, limbs movements, head movements, discussing with gestures, etc.); a high level means standing with significant movements usually indicating intentional actions like sustained location changes by walking, constant trunk movements to stretch/relax the body, etc.

It is extremely important that the three levels do not mean there are only three options for the *M* value. Instead, for a person’s activity intensity, there are three classification probabilities $P_{l}$, $P_{m}$, and $P_{h}$ indicating the possibilities of being viewed as low, moderate, and high level, respectively. Based on $P_{l}$, $P_{m}$, and $P_{h}$, the person’s final *M* is estimated by:

(8) $$M=P_{l}\;\cdot \;M_{l}+P_{m}\;\cdot \;M_{m}+P_{h}\;\cdot \;M_{h}$$

where $M_{l}$, $M_{m}$, and $M_{h}$ are the lower boundary, the middle value, and the upper boundary of a standing person’s *M*, that are, 75 W/m^2^, 125 W/m^2^, and 174 W/m^2^, respectively.

To realize this solution, the classification probabilities $P_{l}$, $P_{m}$, and $P_{h}$ are in need. With only three features describing a person’s activity intensity within a few seconds as the input, a simple and flexible classification model instead of a CNN can be used. So, in this study, several lightweight models are employed and the random forest model works best. The training and testing details are in Section 4.4.

In summary, the proposed *M* estimation method has several advantages: (i) the three explicitly-extracted features can guide the metabolic rate estimation efficiently, considering that the features automatically extracted by a learning method are relatively difficult to anticipate and thus may potentially fail for a specific task; (ii) the three features are really low dimensional, making it possible to use lightweight machine learning classifiers which are flexible to be integrated into the whole system; (iii) the probability-weighted summation (Equation (8)) makes the estimated *M* continuously change in a range, which not only fits the real-life scenario than limited and discrete choices in existing methods but also avoids the very difficult annotation if a regression model is adopted.

## 4. Experiments

In this part, we first introduce the information of the dataset we collected from a multi-person environment, and then the proposed tracking-by-detection module, $I_{cl}$ estimation module, and *M* estimation module are evaluated.

### 4.1. Dataset Information

There is no available public dataset for visual analysis of $I_{cl}$ and *M* in a multi-person environment. We, therefore, collected such a dataset in December 2020 in Denmark. During the collection, two persons were sitting or standing with different types of clothes in a typical office environment where the indoor temperature and humidity were 22 °C and 32%, and they were encouraged to behave naturally. That means, typing the keyboard, texting with cellphones, chatting with each other, reading, stretching the body to relax, and others were captured in the collected videos. The horizontal distance between the camera and persons is around 3.5 meters, and the vertical distance between the camera and the ground is around 2.7 meters. In this way, ten subjects contributed to 114 videos with each video’s length about 2000 frames by using a thermal camera (Xenics Gobi-384-GigE whose sensor size is 384×288).

### 4.2. Evaluation of the Tracking-by-Detection Module

The tracking-by-detection (DeepSort-by-YOLOv5) module needs a well-trained human detector to detect persons in six categories mentioned before in Table 3. To train YOLOv5, from the dataset we sampled one frame every 50 frames for annotation and thus 5263 frames are selected in which each person’s bounding box and category are labeled. These 5263 images are then divided into a training set (4467), validation set (362), and testing set (434) to guarantee that subjects in the testing set never exist in the training set and validation set for a fair evaluation. Additionally, we selected and labeled 832 images from a single-person thermal dataset from [34] to increase the amount and diversity of the training set. The detailed information of the data to train and evaluate YOLOv5 is listed in Table 4. Accordingly, the 15 videos from which the 434 testing images are sampled are used to evaluate the whole DeepSort-by-YOLOv5 framework.

**Figure 6:**
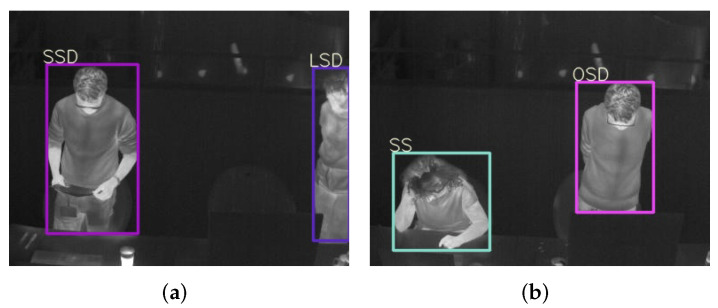
Detection results on two test images with OclStand (OSD) persons in them. (**a**) The right person is wrongly categorized as LongStand (LSD). (**b**) Both persons are detected and categorized correctly.

With a desktop equipped with Windows 10, CUDA (Compute Unified Device Architecture) 10.2, Pytorch 1.7.1, and one NVIDIA 2080Ti GPU (Graphics Processing Unit) card, the YOLOv5m version [41] is finetuned with the learning rate 0.0075 and stops at the 200th epoch at which the training loss is not decreasing any more. Other settings remain the same with the released YOLOv5m. The best model on the validation set is performed on the testing set and then achieves a mAP_50_ (Mean Average Precision) of 89.1% over six categories. Specifically, the AP_50_ rates of LongSit, ShortSit, OclSit, LongStand, ShortStand, and OclStand are 98.8%, 90.0%, 95.5%, 98.5%, 99.5%, and 52.5%, respectively. The AP_50_ drop in OclStand is due to the data imbalance problem. There are less than 300 images having OclStand persons in the training set, and there are only two images having Oclstand persons in the testing set (see Figure 6). In Figure 6, persons with bounding box SSD (ShortStand), SS(ShortSit), and OSD (OclStand) are categorized correctly, while the one with box LSD (LongStand) is categorized wrongly since the person’s sleeve status is unknown and thus should have been recognized as OclStand (OSD).

**Figure 7:**
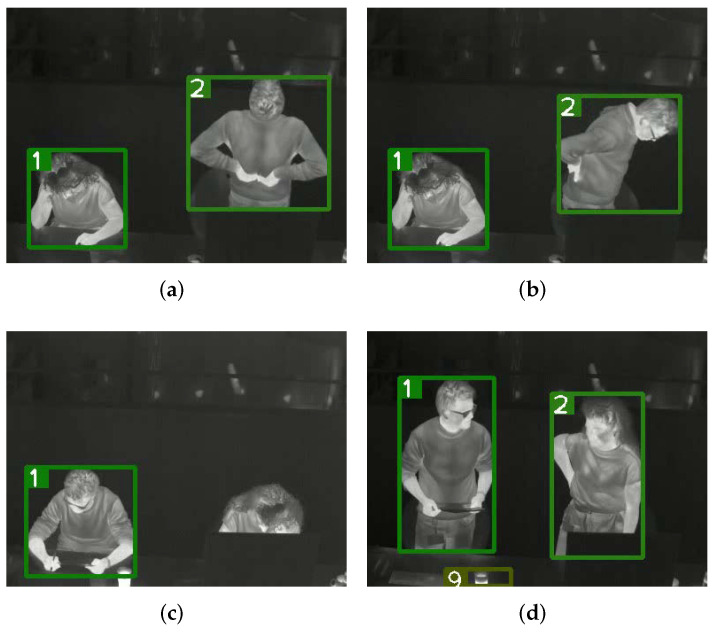
Sampled tracking results on the testing set, no false positive and false negative in (**a**,**b**), one false negativeFN in (**c**), one false positive in (**d**). The numbers indicate tracked ID numbers.

With the same hardware and software platforms, DeepSort-by-YOLOv5 runs on the 15 testing videos without further fine-tuning of the tracker itself. There are a total of 44,077 ground truth persons, 206 false negatives, 16 false positives, and 0 ID-switch in the 15 videos, which achieves an average MOTA (Multiple Object Tracking Accuracy) of 99.5% and the lowest MOTA of an individual video is 93.7%. Figure 7 shows four sampled tracking results. The eight persons from left to right in Figure 7 are in category ShortSit, LongStand, ShortSit, LongStand, ShortSit, OclSit, ShortStand, and ShortStand, respectively. Figure 7a,b are near frames from a video, and both persons are well tracked though the person with ID 2 is moving intensely. The false negative in Figure 7c is because there is no similar situation in the training set that a person is occluded so severely. The mug with hot coffee in Figure 7d has a similar temperature distribution as humans, which leads to the false positive.

In summary, the proposed DeepSort-by-YOLOv5 module achieves a mAP_50_ rate of 89.1% and a MOTA rate of 99.5% on the testing data. As this is the first work on multi-person analysis in terms of clothing and activity status recognition for thermal comfort, a direct comparison with other works is not possible. Instead, we refer to the latest performance of human detection/tracking on other thermal databases as an indirect comparison. Work [51] shows that the mAP_50_ values are from 62.0% to 96.0% on benchmark databases with different difficulties like OSU, KAIST, VOT-TIR2015, etc. Work [68] shows that the MOTA values are from 54.3% to 64.9% with different trackers on SCUT-FIR pedestrian dataset. These reference results indicate that our results are good enough and thus the proposed method can be included in a real application.

### 4.3. Evaluation of the $I_{cl}$ Estimation Module

The $I_{cl}$ estimation closely depends on the skin temperature $T_{s}$ and clothes temperature $T_{c}$ acquisition, which is bridged by the localization of skin region $R_{s}$ and clothing-covered region $R_{c}$ via OpenPose. Therefore, this evaluation first looks at the efficacy of applying OpenPose to our dataset.

4901 images are used to examine OpenPose’s performance. These 4901 images come from the 5263 annotated images for YOLOv5 but do not include the images where persons are wearing masks due to coronavirus restrictions. Such an evaluation set is evenly sampled from the 114 collected videos, guaranteeing comprehensiveness and fairness. The evaluation protocols are: (i) the OpenPose tool is not finetuned with our thermal dataset, and the confidence threshold is set as 0.6 as mentioned in Section 3.2; (ii) only these key points that influence $R_{s}$ and $R_{c}$ localization are checked, i.e., nose, shoulders, elbows, and wrists; (iii) any frame with even only one wrongly detected key body point is counted as one error frame, to make the evaluation strict and conservative.

**Figure 8:**
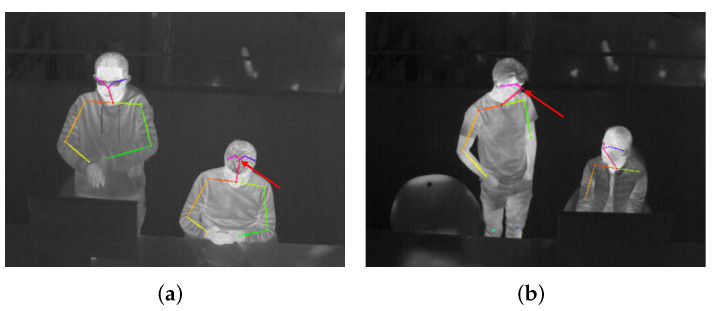
Two representative error frames with red arrows pointing to the wrongly detected noses. (**a**) Nose is detected in the hair region. (**b**) Nose is detected in the background region.

After a frame-by-frame check, there are 187 error frames out of the whole 4901 frames, indicating an accuracy of 96.2%. We found that there are two types of representative errors—nose detected in the hair region due to a lowering head (Figure 8a) and nose detected in the background region due to a turned side face (Figure 8b). The good point is that with the average computation within a few minutes to get $T_{s}$ and $T_{c}$, the influence of these errors can be eliminated effectively, and of course, a higher confidence threshold can further reduce such errors if needed.

Therefore, the efficacy of applying OpenPose to our multi-person thermal scenario to locate $R_{s}$ and $R_{c}$ is verified. The performance surpasses that of applying OpenPose to a controlled single-person thermal environment [35] and applying OpenPose to RGB MPII dataset [69], further proving the feasibility of our strategy relying on OpenPose.

**Figure 9:**
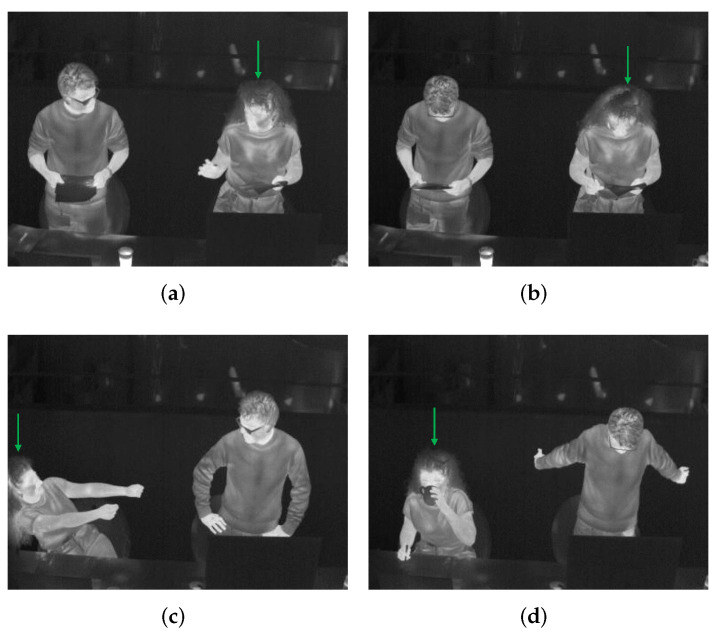
The female to be researched is pointed by the green arrow, (**a**) chatting, (**b**) reading, (**c**) chatting with gestures, (**d**) drinking.

Based on the above acquired $R_{s}$ and $R_{c}$, here we calculate the $T_{s}$ and $T_{c}$, and then estimate the $I_{cl}$ value. Since an individual $I_{cl}$ estimation also involves the human tracking part, we use the testing videos for the tracking module to evaluate this $I_{cl}$ estimation module too. From the testing videos, a female wearing a lightweight T-shirt is acting as the subject to be researched, because there is an available reference for her clothes type in the ISO tables so that we can make a comparison. And thus, two videos including various situations where the female is sitting, standing, reading, writing, typing the keyboard, chatting, and drinking coffee (some frames are shown in Figure 9) go through our methodology pipeline to get her $I_{cl}$. In one video consisting of 1477 frames (70 seconds), 3326 skin points and 2849 clothes points are detected for the female, from which the $T_{s}$ and $T_{c}$ are calculated as 34.67 °C and 33.32 °C, respectively. Together with the $T_{o}$ as 24.96 °C, the female’s $I_{cl}$ is estimated as 0.1220 clo. In the other video of 1536 frames (73 seconds), 2496 skin points and 2502 clothes points are detected for the female; the resultant $T_{s}$ is 34.73 °C and $T_{c}$ is 33.48 °C; together with the $T_{o}$ as 25.58 °C, the female’s $I_{cl}$ is estimated as 0.1182 clo.

From above calculation, we find that: (i) within a time period like more than 1 minute, the accumulated detected points in $R_{s}$ and $R_{c}$ are way enough for an accurate $T_{s}$ and $T_{c}$ calculation as the potential noises can be filtered out efficiently; (ii) the estimated $I_{cl}$ values of 0.1220 clo and 0.1182 clo are quite similar, revealing the stability and robustness of the method; (iii) the reference value of the female’s $I_{cl}$ is 0.09 clo to 0.15 clo from Table B.1 in ISO 9920 [10], showing the consistency of our method with the international standards, and proving the feasibility of the proposed method.

### 4.4. Evaluation of the M Estimation Module

This subsection evaluates the effectiveness of the *M* estimation based on the three extracted vision features, specifically for a standing person. As this estimation is a probability-weighted summation, measuring the accuracy of the classifier is the key.

Therefore, by dividing the 114 collected videos into small clips of 10 seconds and then extracting the three vision features for each standing person in these clips, 315 sets of the three features are used as the training data to help the classifier learn the ability to category each person’s activity intensity into low, moderate, or high level, and another 68 sets are used as the testing data to evaluate the classifier’s performance.

During the phase of preparing the training and testing data—annotating a standing person’s activity intensity level, we met another dilemma that frequently happens in the real world—there are always the situations where a person’s movement is mixed with transient, lasting, mild, or intensive movements within a short period which makes it very difficult to label the intensity level. Therefore, these difficult cases are not included in the training/testing sets to not confuse the classifier. From the positive side, this situation further indicates the strength of our probability-weighted summation strategy that makes the estimated *M* a continuous value.

To avoid being one-sided, three widely-used classifiers—KNN (K-NearestNeighbor), SVM (Support Vector Machine), and RF (Random Forest) are used. The parameters and performances of the three classifiers are listed in Table 5, in which each parameter is tuned by grid searching using the training data and the meaning of each parameter is explained in the scikit-learn library [70]. These accuracy values in Table 5 prove that the three features are good representations of a person’s activity intensity, and thus the *M* estimation from them by a classifier’s probability-weighted summation is also reasonable. And then we decide to use RF as the classifier for *M* estimation due to its best performance on the testing data.

**Figure 10:**
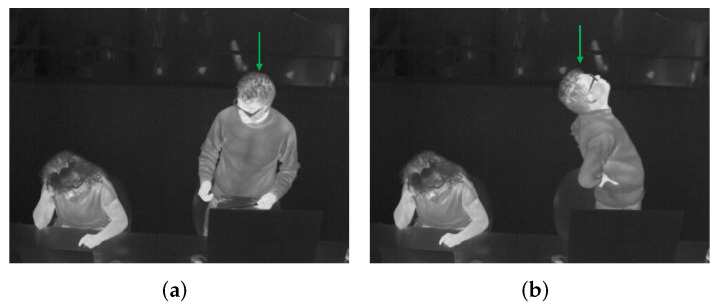
The standing person to be researched for *M* estimation is pointed by the green arrow. (**a**) normally chatting but with many gestures, (**b**) stretching body like doing Pilates.

Based on RF’s classification probabilities $P_{l}$, $P_{m}$, and $P_{h}$, by Equation (8), the *M* values of a same standing person with two totally different activity intensities are estimated. The person is shown in Figure 10, in which Figure 10a is a frame from a clip where the standing person is normally chatting with many gestures, and Figure 10b is a frame from another clip where the standing person is stretching his body like doing Pilates. For them, our method outputs the estimated *M* values of 99 W/m^2^ and 170 W/m^2^, respectively, which are very similar to the reference values of 104 W/m^2^ (CODE 09050 in [67]) and 174 W/m^2^ (CODE 02105 in [67]), further proving the feasibility and usability of the proposed *M* estimation module.

### 4.5. Application in Thermal Comfort Assessment

From all the above evaluations, the proposed method indeed has the ability to estimate individual $I_{cl}$ and *M* across time for each person in a room. With these two dynamic personal factors and the other four environmental factors easily measured from sensors, a thermal comfort model like Fanger’s model [6,7] can calculate individual thermal comfort sensation to see if the person feels hot, cold, or satisfied with the indoor environment. Although occupants may have different thermal feelings at the same time, by regulating the indoor microclimate in separate local regions, it is possible to achieve varied thermal conditions that respond to the different subjective thermal states. Moreover, the used thermal camera instead of an RGB camera, the computation in a local device, and the erasing function of captured image information as long as $I_{cl}$ and *M* are estimated will make the whole processing pipeline privacy-friendly.

## 5. Conclusions and Future Work

This paper proposes a contactless method to estimate each person’s clothing insulation rate $I_{cl}$ and metabolic rate *M* dynamically by use of a thermal camera, in an uncontrolled multi-person indoor environment.

Specifically, the method composes of a tracking-by-detection (DeepSort-by-YOLOv5) module to track each person and recognize his or her clothing status and key posture simultaneous, a key body points detection module to measure the skin temperature and clothes temperature for $I_{cl}$ estimation, and a random forest classifier module to categorize each individual’s activity intensity into different levels for *M* estimation. All three modules are evaluated with a new multi-person thermal dataset, verifying that the methodology is robust to be applied in real-life applications for individual thermal comfort assessment.

The future work will be to include this research into such an application to facilitate thermal comfort control systems for lower energy waste and higher working comfort in an office building.

## Figures and Tables

**Table 1:** Insulation values of various typical garments [10].

Garment		$I_{\mathrm{cl}}$ (clo)
Underwear	Singlet	0.04
	T-shirt	0.09
	Shirts with long sleeves	0.12
Shirts, blouses	Short sleeves	0.15
	Lightweight, long sleeves	0.2
	Normal, long sleeves	0.25

**Table 2:** Metabolic rates of typical activities [8].

Activity	*M* (W/m^2^)
Reclining	46
Seated, relaxed	58
Sedentary activity	70
Standing, light activity	93
Standing, medium activity	116
Walking on level ground:	
2 km/h	110
3 km/h	140
4 km/h	165
5 km/h	200

**Table 3:** Persons in six categories.

Category	Meaning
LongSit	Long-sleeved clothes, sitting
ShortSit	Short-sleeved clothes, sitting
OclSit	Difficult to predict clothes type due to occlusion, sitting
LongStand	Long-sleeved clothes, standing
ShortStand	Short-sleeved clothes, standing
OclStand	Difficult to predict clothes type due to occlusion, standing

**Table 4:** Detailed information of the data to train and evaluate YOLOv5.

	Number of Images	Number of Persons					
		LongSit	ShortSit	OclSit	LongStand	ShortStand	OclStand
Training	5299	2099	1615	828	2280	2735	254
Validation	362	172	29	274	140	100	9
Testing	434	22	157	92	149	443	2

**Table 5:** The parameters and performances of the used three classifiers.

	Parameters	Training Accuracy	Testing Accuracy
KNN	metric=‘manhattan’, weights= ‘distance’, n_neighbors=13	100%	92.7%
SVM	C=50, kernel=‘rbf’, gamma=‘scale’	83.5%	88.2%
RF	max_depth=2, random_state=0	95.6%	95.6%

## Data Availability

To protect the facial information of some participators, only a partial dataset is available on request from the corresponding author.

## Abbreviations

The following abbreviations are used in this manuscript:

mAP	Mean Average Precision
MOTA	Multiple Object Tracking Accuracy
HVAC	Heaters, ventilation, and air conditioners
CNN	Convolutional Neural Network
ISO	International Organization for Standardization
R-CNN	Region-based Convolutional Neural Network
YOLO	You Only Look Once
FPN	Feature Pyramid Network
RGB	Red Green Blue
SORT	Simple Online and Realtime Tracking
DeepSort	Simple Online and Realtime Tracking with a Deep Association Metric
CDA_DDAL	Confidence-based Data Association and Discriminative Deep Appearance Learning
ID	Identity
PANet	Path Aggregation Network
FN	False Negative
FP	False Positive
TV-L1	Total Variation L1 Norm
CBE	Center for the Built Environment
CUDA	Compute Unified Device Architecture
GPU	Graphics Processing Unit
KNN	K-NearestNeighbor
SVM	Support Vector Machine
RF	Random Forest
